# Minimally invasive delivery of therapeutic agents by hydrogel injection into the pericardial cavity for cardiac repair

**DOI:** 10.1038/s41467-021-21682-7

**Published:** 2021-03-03

**Authors:** Dashuai Zhu, Zhenhua Li, Ke Huang, Thomas G. Caranasos, Joseph S. Rossi, Ke Cheng

**Affiliations:** 1 Department of Molecular Biomedical Sciences and Comparative Medicine Institute, North Carolina State University, Raleigh, NC USA; 2 Joint Department of Biomedical Engineering, University of North Carolina at Chapel Hill & North Carolina State University, Raleigh, NC USA; 3 Division of Cardiothoracic Surgery, University of North Carolina at Chapel Hill, Chapel Hill, NC USA; 4 Division of Cardiology, University of North Carolina at Chapel Hill, Chapel Hill, NC USA

**Keywords:** Cardiology, Translational research

## Abstract

Cardiac patches are an effective way to deliver therapeutics to the heart. However, such procedures are normally invasive and difficult to perform. Here, we develop and test a method to utilize the pericardial cavity as a natural “mold” for in situ cardiac patch formation after intrapericardial injection of therapeutics in biocompatible hydrogels. In rodent models of myocardial infarction, we demonstrate that intrapericardial injection is an effective and safe method to deliver hydrogels containing induced pluripotent stem cells-derived cardiac progenitor cells or mesenchymal stem cells-derived exosomes. After injection, the hydrogels form a cardiac patch-like structure in the pericardial cavity, mitigating immune response and increasing the cardiac retention of the therapeutics. With robust cardiovascular repair and stimulation of epicardium-derived cells, the delivered therapeutics mitigate cardiac remodeling and improve cardiac functions post myocardial infarction. Furthermore, we demonstrate the feasibility of minimally-invasive intrapericardial injection in a clinically-relevant porcine model. Collectively, our study establishes intrapericardial injection as a safe and effective method to deliver therapeutic-bearing hydrogels to the heart for cardiac repair.

## Abstract (web summary)

Current routes to deliver therapeutics to the heart are normally invasive or difficult to perform. Here the authors develop intrapericardial injection as an efficient, easy-to-perform and minimally invasive method to deliver therapeutics for cardiac repair.

## Introduction

Cardiovascular diseases remain the number 1 killer in western societies^1^. In a major heart attack, or myocardial infarction (MI), a patient can lose about one billion healthy cardiomyocytes^2^. The ischemic area will be infiltrated by inflammatory cells and later on replaced by cardiac fibrosis^3^. Once advanced heart failure occurs, a heart transplantation is the only option^4^. Regenerative therapies using live cells, proteins, and nuclei acids aims at altering the trajectory of adverse heart remodeling and promoting de novo cardiac repair^5–8^. However, it is difficult to deliver therapeutics to the heart, with high efficiency and low invasiveness and cost^9^. A cardiac patch can effectively deliver therapeutics to the heart^10–12^, yet such procedures normally require open chest surgery.

The heart is an organ deeply embedded in the thoracic cavity. There are several ways to delivery therapeutics to the heart^13^. Intravenous (IV) injection is quite safe and convenient (no anesthesia is needed), yet has a poor cardiac retention of the therapeutics^14^. On the contrast, intramyocardial (IM) injection, i.e. direct injection of therapeutics into the myocardium, can get a sizable amount of drug into the heart, but usually requires open chest surgery^5^ or sophisticated systems such as NOGA mapping coupled with transendocardial injection^15^. Intracoronary injection can be readily performed by an interventional cardiologist^16^ with local anesthesia. However, cardiac retention is not ideal, and is only slightly better than IV injection^17^. Recently, tissue engineering approaches have shed some light on improving biodistribution in the heart^18,19^. Laying a cardiac patch on the surface of the heart can normally generate the greatest cardiac retention^20,21^. However, such procedures are difficult to perform, quite invasive, and not suitable for patients with mild-to-moderate heart diseases. In addition, the therapeutics in the patch can leak into the thoracic cavity and/or cause adhesion to the thoracic wall^22^. We sought to develop a method that can deliver a therapeutic cardiac patch to the heart in a minimally invasive fashion and with optimal biodistribution in the heart. We turned our attention to the pericardium. Pericardium is a double-walled sac that gives protection against infection and provides the lubrication for the heart. This space between the serous and fibrous pericardium is called the pericardial cavity^23^. The pericardial cavity is filled with pericardial fluid. We reason that the pericardial cavity is a perfect space to serve as a natural “mold” for injectable hydrogels to form a uniform cardiac patch covering the entire heart. Indeed, intrapericardial (iPC) procedures are normally performed for epicardial catheter mapping and ablation^24^ or for other diagnosis purposes^25^.

In this study, we hypothesize that we can deliver therapeutics in a biocompatible hydrogel to the pericardial cavity to form a cardiac patch in situ, without the need of any suture or glue. After injection, hydrogel degradation will lead to sustained release of therapeutics into the myocardium^15^ for cardiac repair. This method is also highly translatable. With the aid of a fluoroscope, iPC access and injection in humans can be performed with only one incision on the chest under local anesthesia^26^.

## Results

### Feasibility of iPC injection

All animal studies were approved by the Institutional Animal Usage and Care Committee of North Caroloina State University. We first demonstrated that iPC injection can be performed in mice and rats with open chest surgery (Supplementary Movies 1 and 2), and mini-invasively in pigs with two small incisions (one for the injection needle and the other for the camera probe) on the chest wall (Supplementary Movie 3). Next, we tested the efficacy and safety of iPC injection for cardiac repair, using induced pluripotent stem cell-derived cardiac progenitors (iPS-CPCs) and mesenchymal stem cell (MSC)-derived exosomes as our model therapeutics.

### iPC injection of pluripotent stem cells causes less immune responses

**Fig. 1:**
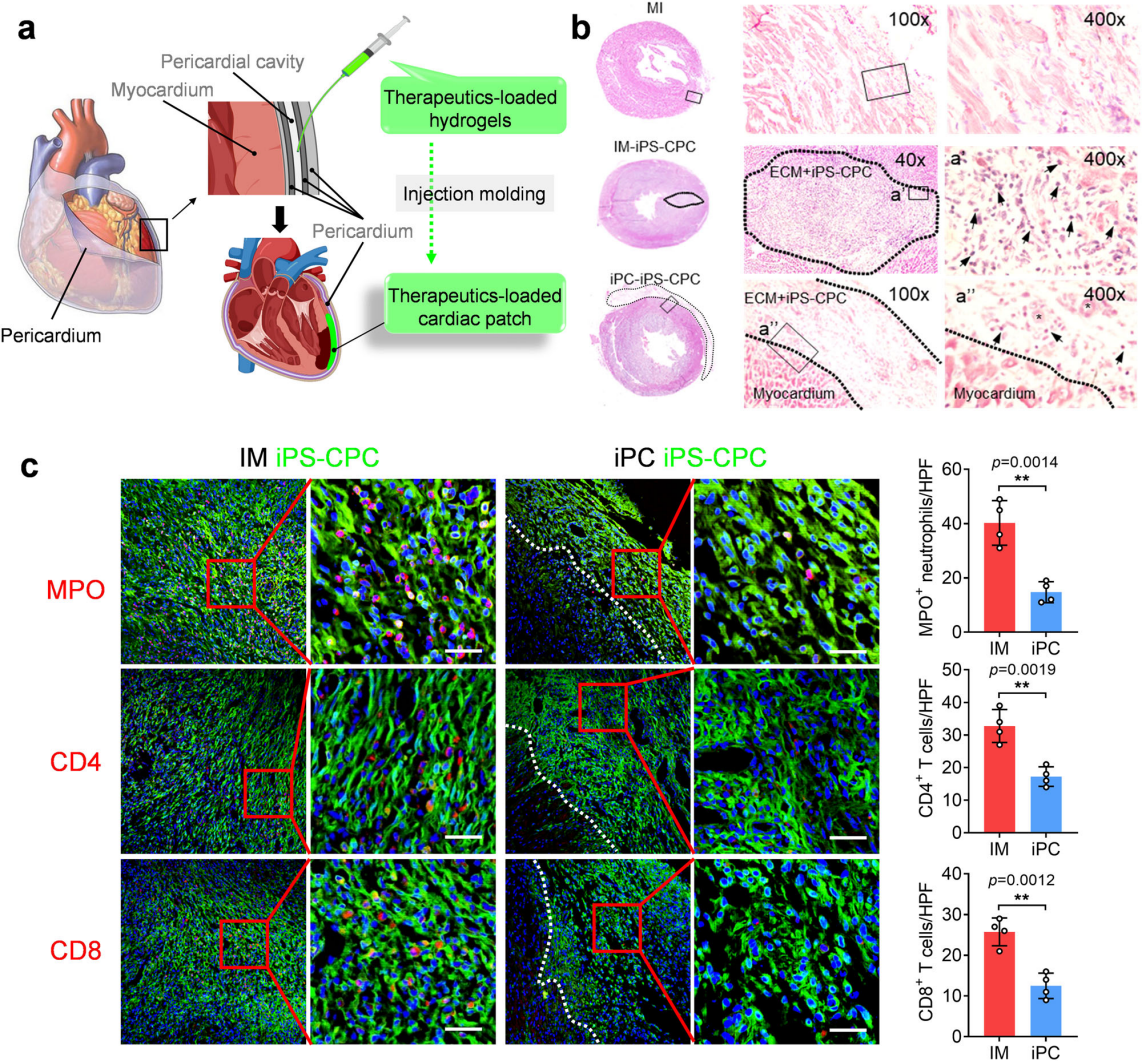
iPC injection mitigates immune response as compared to IM injection in rats. **a** Schematic illustration of iPC injection and in situ formation of cardiac patch. **b** H&E staining showing the formation of a cardiac patch 7 days after iPC injection in the iPC-iPS-CPC group (dash lines delineate the injected cells). Intramyocardial (IM) injection of iPS-CPCs in ECM hydrogel caused massive infiltration of immune cells (arrows). iPC injection reduced immune cell infiltration and promoted regional formation of blood vessels (asterisks) in the patch. **c** Representative microscopic images showing the presence of neutrophils, CD4 and CD8 T cells, and corresponding quantitation. Scale bar, 60 μm. Data were shown as mean ± SD, *n* = 4 animals per group. Comparison of two groups was performed with the unpaired, two-tailed Student’s *t*-test, **p < 0.01. Source data are provided in a Source Data file. MI myocardial infarction, iPC intrapericardial injection, IM intramyocardium injection, iPS-CPC induced pluripotent stem cell-derived cardiac progenitor cell, ECM extracellular matrix, MPO myeloperoxidase, CD cluster of differentiation, HPF high-power field.

Our first study involves iPC injection of iPS-CPCs in an injectable decellularized extracellular matrix (ECM) hydrogel made from porcine heart. We tested the therapy in a rat model of MI (Fig. 1). Pig heart-derived ECM was characterized (Supplementary Fig. 1). Biocompatibility was confirmed after direct injection into the pericardial cavity (Supplementary Fig. 2). We also confirmed the ability of iPS-CPCs for cardiac differentiation in vitro (Supplementary Fig. 3). Emerging evidence supports the beneficial effects of induced pluripotent stem cell (iPSC) therapy on ischemic heart diseases by both direct differentiation and paracrine effects^27–29^. However, IM injection of iPS cells can potentially lead to teratoma formaion, immune rejection, and arrhythmia^28^. We showed that iPC injection of iPS-CPCs in ECM hydrogel formed a cardiac patch-like structure in the infarct (Fig. 1a, b). Moreover, iPC injection attenuated immune response, which was evident in the IM injection group (Fig. 1b, c). Infiltration of neutrophils and T cells was observed in IM-injected hearts, but was negligible in the iPC-injected animals (Fig. 1b, c).

### iPC delivery of stem cells leads to cardiac regeneration and repair

**Fig. 2:**
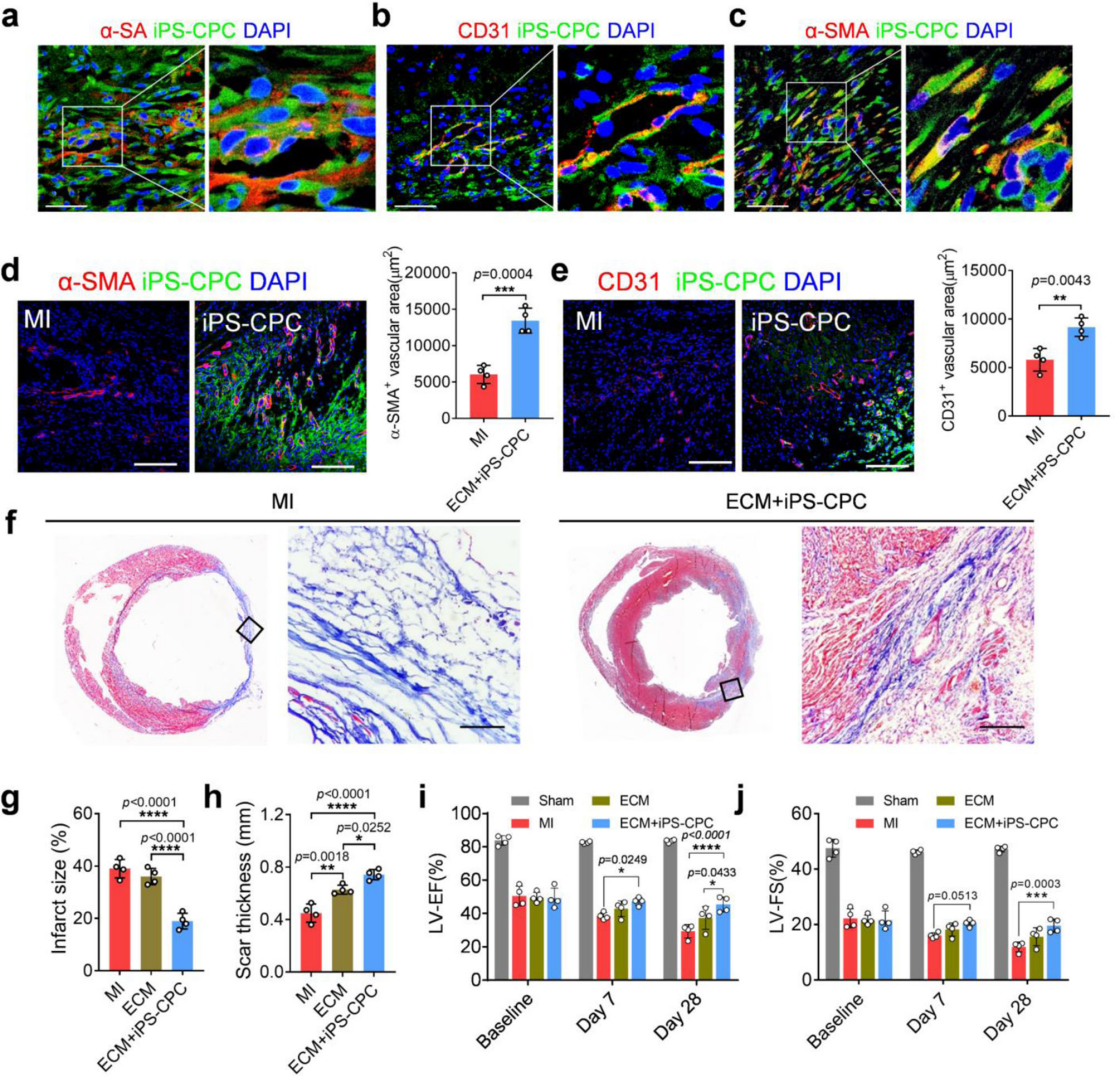
iPC-injected iPS-CPCs contribute to cardiac regeneration and repair in a rat model of MI. **a**–**c** Hearts were collected 7 days after injection, and in vivo differentiation of iPS-CPCs into cardiac and vascular lineages after iPC injection were detected. Scale bar, 60 μm. **d**, **e** Representative confocal microscopic images showing immunostaining of α-SMA and CD31 in the hearts 7 days after injection and corresponding quantification. Scale bar, 60 μm. Data were shown as mean ± SD, *n* = 4 animals per group. Comparison of two groups was performed with the unpaired, two-tailed Student’s *t*-test, **p < 0.01, ***p < 0.001. **f** Representative heart sections with Masson’s trichrome staining (red = healthy tissue; blue = scar) 4 weeks after treatment. Scale bar, 60 μm. From the Masson’s trichrome-stained images, infarct size (**g**) and LV wall thickness (**h**) were quantified. Data were shown as mean ± SD, *n* = 4 animals per group. Comparison of three groups was performed with the two-tailed one-way ANOVA, *p < 0.05, **p < 0.01, ****p < 0.0001. **i**, **j** iPC injection of iPS-CPCs in ECM improved cardiac functions. Data were shown as mean ± SD, *n* = 4 animals per group. Statistical analysis was performed with two-tailed two-way ANOVA, *p < 0.05, ***p < 0.001, ****p < 0.0001. **i** *p* = 0.0249 (ECM+iPS-CPC vs MI, day 7), *p* < 0.0001 (ECM+iPS-CPC vs MI, day 28), *p* = 0.0508 (ECM vs MI, day 28), *p* = 0.0433 (ECM+iPS-CPC vs ECM, day 28). **j** *p* = 0.0513 (ECM+iPS-CPC vs MI, day 7), *p* = 0.0003 (ECM+iPS-CPC vs MI, day 28), *p* = 0.1384 (ECM vs MI, day 28), *p* = 0.0944 (ECM+iPS-CPC vs ECM, day 28). Source data are provided as a Source Data file. MI myocardial infarction, iPC intrapericardial injection, iPS-CPC induced pluripotent stem cell-derived cardiac progenitor cell, ECM extracellular matrix, α-SA sarcomeric α-actinin, α-SMA smooth muscle α-actin, LVEF left ventricular ejection fraction, LVFS left ventricular fraction shortening.

Immunostaining confirmed that iPC-injected iPS-CPCs differentiated into cardiomyocytes, smooth muscle cells, and endothelial cells (Fig. 2a–c and Supplementary Movie 4) in the post-MI heart. Such direct differentiation was also accompanied by indirect paracrine mechanisms of repair. iPC injection of iPS-CPCs promoted angiogenesis (Fig. 2d, e) and reduced infarct size (Fig. 2f, g). Consistent with the improved cardiac morphology (Fig. 2h and Supplementary Fig. 4), cardiac function was protected by iPS-CPC treatment (Fig. 2i, j, Supplementary Fig. 5 and Supplementary Table 1). Collectively, those datasets suggested that iPC delivery of iPS-CPCs in biomaterials is safe and effective for cardiac repair in a rodent model of MI.

### iPC injection in hydrogel enhances MSC exosome retention in the heart

**Fig. 3:**
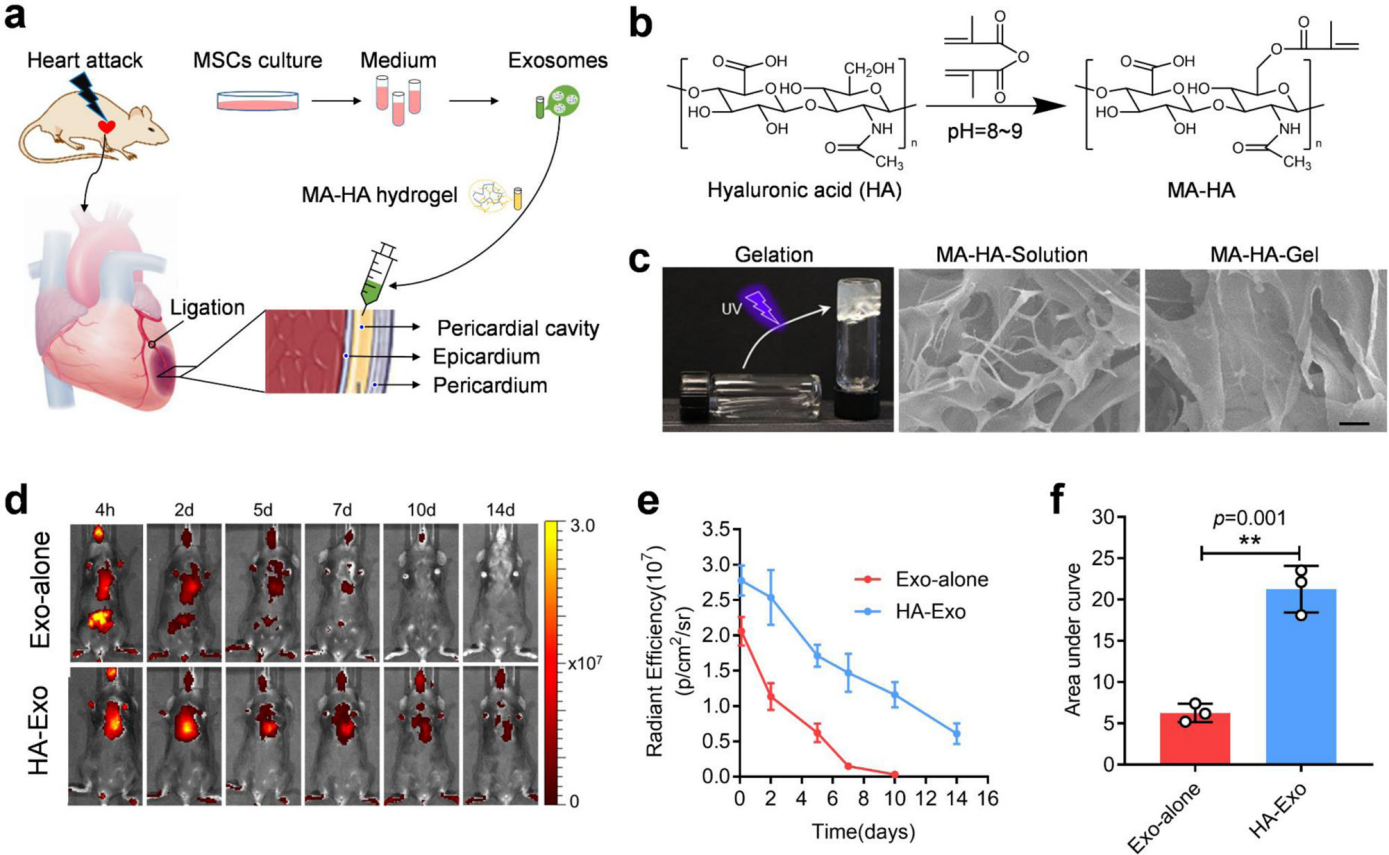
iPC injection of MSC exosomes in MA–HA hydrogel in a mouse model of acute MI. **a** Schematic showing intrapericardial delivery of exosomes for MI therapy. **b** Synthesis of MA–HA hydrogel. **c** In vitro gelation of MA–HA hydrogel under UV irradiation and SEM images of MA–HA hydrogel before and after gelation. Scale bar, 50 μm. The gelling process was repeated three times independently with the similar results. **d** Fluorescent imaging of mice after iPC injection of DiD-labelled exosomes with or without MA–HA hydrogel. **e** Quantitative data of fluorescence intensity. Data were shown as mean ± SD, *n* = 3 animals per group. **f** The area under curve was determined. Data were shown as mean ± SD, *n* = 3 animals per group. Comparison of two groups was performed with the unpaired, two-tailed Student’s *t*-test, ***p < 0.001. Source data are provided as a Source Data file. MSCs mesenchymal stem cells, MA–HA methacrylated hyaluronic acid, Exo exosomes.

Our second study involves iPC delivery of therapeutic exosomes in hyaluronic acid (HA) hydrogel in a mouse model of MI (Fig. 3a). Exosomes are 30–150 nm extracellular vesicles secreted by essentially all cell types^30^. Exosomes derived from MSCs are promising therapeutic agents in cardiac repair^31^. It is difficult to deliver exosomes directly to the heart. Methacrylic anhydride (MA)–HA hydrogel was synthesized by crosslinking MA with HA to prepare a UV-sensitive hydrogel. Scanning electron microscope imaging revealed the ultrastructure of the gel (Fig. 3b, c and Supplementary Fig. 6a). Exosomes were derived from human MSCs using the ultracentrifugation method and TEM imaging was performed to show exosome morphology (Supplementary Fig. 6b). iPC injection resulted in a nice cardiac retention of exosomes and injection in hydrogel further prolonged the release of exosomes into the heart (Fig. 3d–f).

### iPC delivery of exosomes promotes cardiac repair after MI

**Fig. 4:**
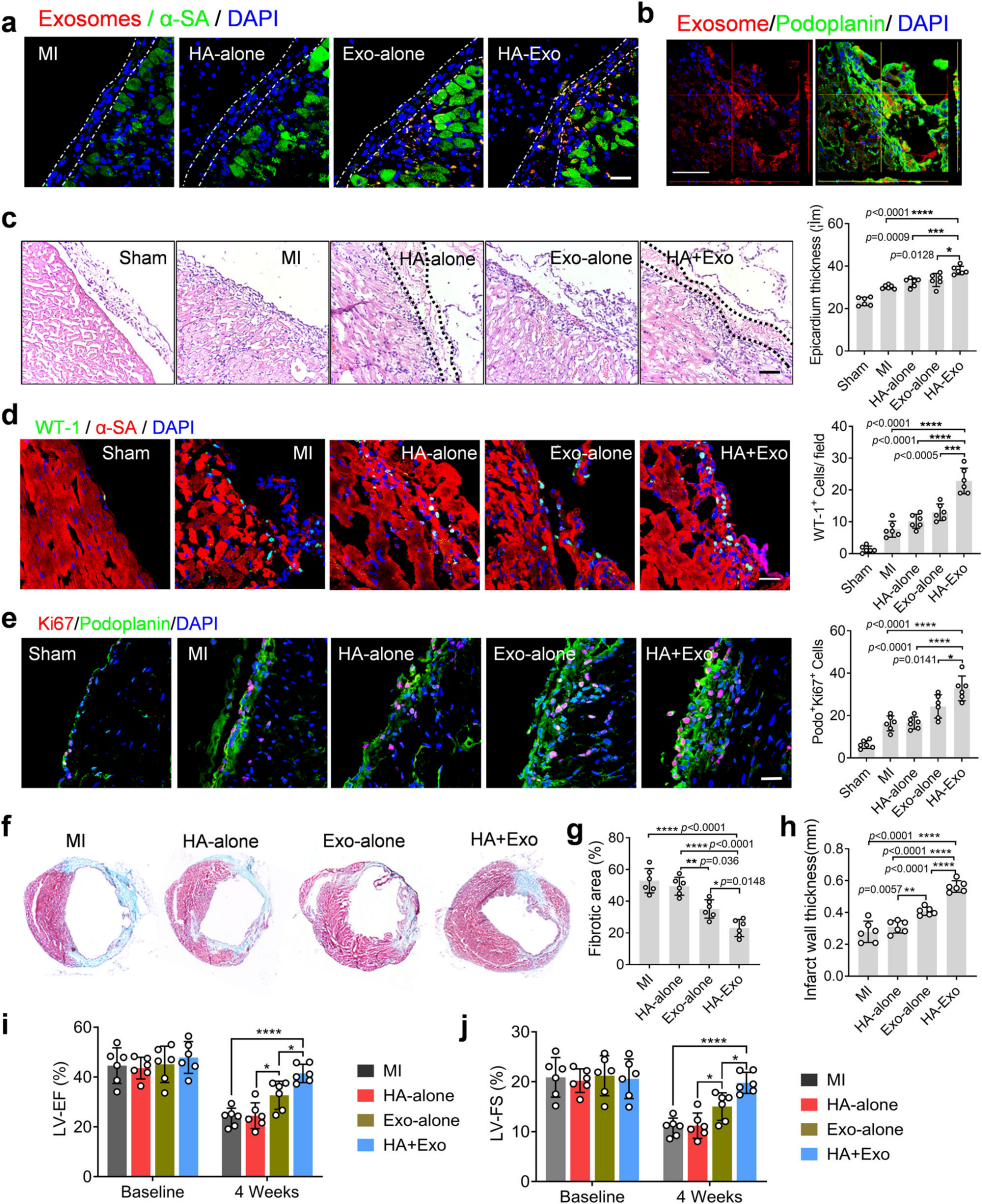
iPC delivery of exosomes stimulated epicardium-derived repair in a mouse model of MI. **a**–**e** Three days after iPC injection, hearts were collected for histological analysis. **a** Myocardial distribution of exosomes after iPC injection. **b** Z-Stack microscopic images showing the uptake of exosomes by epicardial cells, labeled by podoplanin. **c** H&E staining showing the epicardial spreading of HA hydrogel after iPC injection. The thickness of epicardium layer was determined accordingly. **d** iPC injection of exosomes stimulated accumulation of WT-1-positive epicardium-derived progenitor cells (EPDCs). **e** Epicardial cell proliferation was detected by co-localization of Ki67 (a cell proliferation marker) and podoplanin. The numbers of Ki67/podoplanin double-positive cells were counted. **f** Masson trichrome staining was performed 4 weeks after MI, and **g** the fibrotic area as well as **h** the infarct wall thickness was quantified. **a**–**e** Scale bar, 60 μm. **c**–**e**, **g**, **h** Data were shown as mean ± SD, *n* = 6 animals per group. Comparison of multiple groups was performed with the two-tailed one-way ANOVA, *p < 0.05, **p < 0.01, ***p < 0.001, ****p < 0.001. **i**, **j** Echocardiography measurement of cardiac function, including left ventricular ejection fraction (LVEF) and fractional shortening (LVFS) after various treatment. Data are expressed as mean ± SD, *n* = 6 animals per group. Statistical analysis was performed with two-tailed two-way ANOVA, *p < 0.05, ****p < 0.0001. **i** *p* = 0.0443 (Exo-alone vs MI, 4 weeks), *p* < 0.0001 (HA-Exo vs MI, HA-Exo vs HA-alone, 4 weeks), *p* = 0.0447 (HA-Exo vs Exo-alone, 4 weeks). **j** *p* = 0.029 (Exo-alone vs MI, 4 weeks), *p* < 0.0001 (HA-Exo vs MI, HA-Exo vs HA-alone, 4 weeks), *p* = 0.0456 (Exo-alone vs HA-alone, 4 weeks), and *p* = 0.0108 (HA-Exo vs HA-Exo, 4 weeks). Source data are provided as a Source Data file.

We confirmed the uptake of exosomes by epicardial cells (Fig. 4a, b and Supplementary Movie 5) and the spreading of HA hydrogel to form a cardiac patch in the pericardial cavity (Fig. 4c). iPC injection of exosomes increased the thickness of epicardium (Fig. 4c). iPC injection of MSC exosomes promoted the proliferation and differentiation of epicardial-derived cells (Fig. 4d, e and Supplementary Fig. 7). Moreover, a notable accumulation of exosomes in the mediastinal lymph node was detected (Supplementary Fig. 8). Masson’s trichrome staining revealed that iPC injection of HA + Exo reduced fibrotic area in the post-MI heart (Fig. 4f, g). In addition, there was a reduction of apoptotic cells in the HA + Exo-treated hearts (Supplementary Fig. 9). Consistent with the improved cardiac morphology (Fig. 4f, h), echocardiography measurement demonstrated that iPC injection of HA + Exo therapy boosted cardiac functions (Fig. 4i, j). Furthermore, long-term follow up indicated that iPC injection of exosomes with HA hydrogel improved cardiac morphology and suppressed transition to heart failure (Supplementary Fig. 10). Collectively, those datasets suggested that iPC delivery of therapeutic exosomes in biomaterials is safe and effective for cardiac repair.

### Minimally invasive iPC injection in pigs

**Fig. 5:**
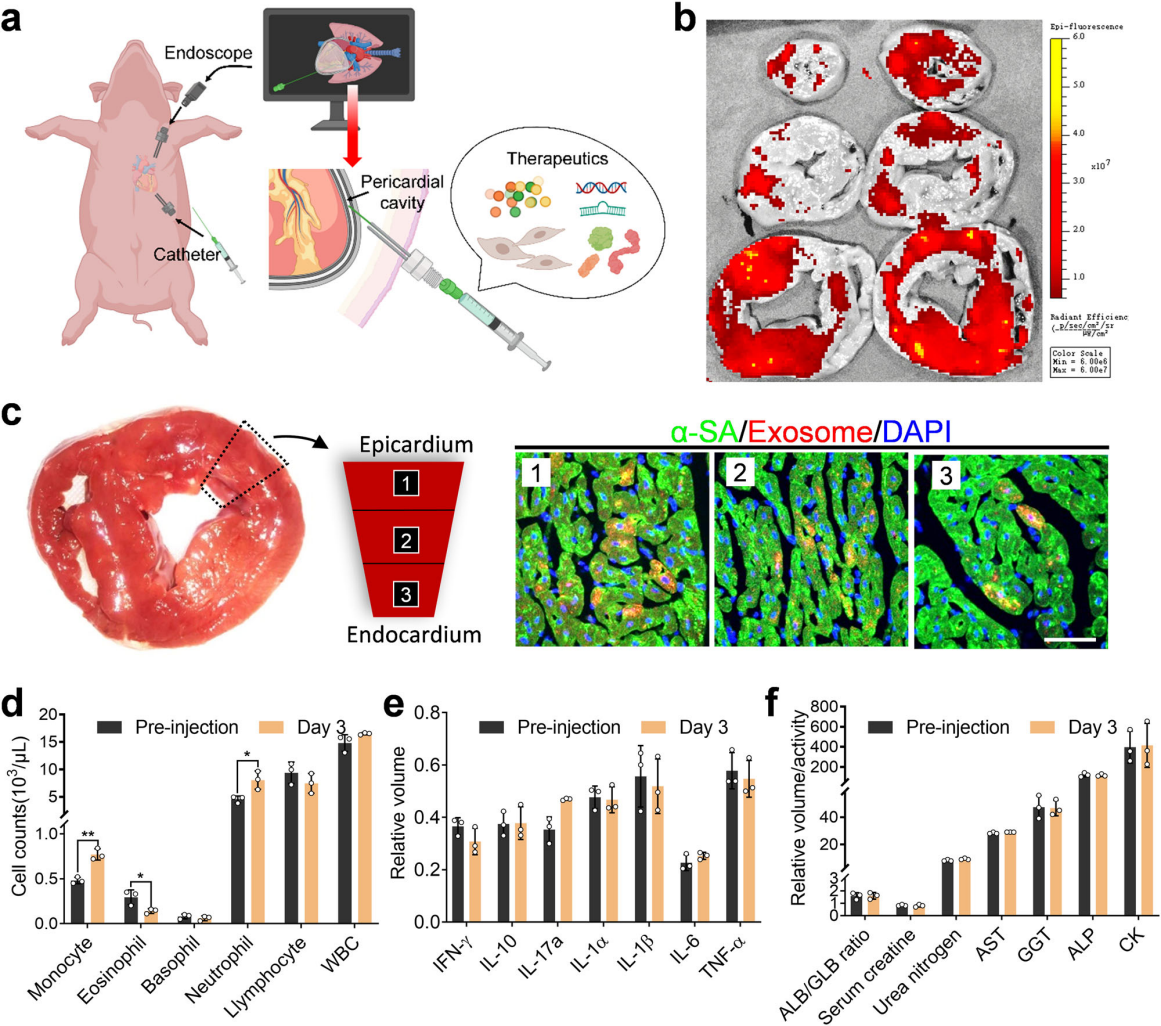
Minimally invasive iPC injection of therapeutics in pigs. **a** Schematic illustration of minimally invasive delivery of therapeutics into pericardial cavity with the aid of endoscope in pigs. **b** Representative ex vivo imaging of pig hearts 3 days after intrapericardial injection of exosomes. **c** Confocal microscopy images showing uptake of exosomes by cardiomyocytes 3 days after iPC injection. Scale bar, 60 μm. This study was repeated in three pigs and similar results were obtained. **d** Analysis of blood cells, **e** inflammatory cytokines in pericardial fluid, and **f** serum chemistry on hepatic, renal, and cardiac functions. Data are expressed as mean ± SD, *n* = 3 animals. Comparison of two groups was performed with the unpaired, two-tailed Student’s *t*-test, *p < 0.05. Monocyte: *p* = 0.0022; eosinophil: *p* = 0.0362, neutrophil: *p* = 0.0268. Source data are provided as a Source Data file. WBC white blood cells, AST aspartate aminotransferase, GGT γ-glutamyl transferase, ALP alkaline phosphatase, CK creatine kinase, IL interleukin, TNF-α tumor necrosis factor-α.

Our third study tested the feasibility of iPC injection of therapeutics in a porcine model. The injection was enabled by two small incisions on the chest wall, one for the injection catheter and the other for the endoscope (Fig. 5a, Supplementary Fig. 11 and Supplementary Movie 3). We used HA + Exo as the model therapy here. Ex vivo fluorescent imaging revealed that iPC injection led to a sizable exosome retention on the heart (Fig. 5b). Histology further confirmed the release and uptake of exosomes by cardiomyocytes, in a wide range from the epicardium to the endocardium (Fig. 5c). We further confirmed the safety of iPC injection in pigs. Three days after the injection, a slight change of cell counts of monocytes, eosinophils, and neutrophils in the blood was observed, which could be caused by the surgery procedures (Fig. 5d), since we didn’t see any difference in the inflammation assay performed on pericardial fluid (Fig. 5e and Supplementary Fig. 12). In addition, there was no change observed in blood chemistry indicators (Fig. 5f). Moreover, iPC injection is feasible in humans as demonstrated by LARIAT procedures^32^, in which the outside catheter is intrapericardially introduced with only one small incision. During such procedures, iPC injection can be performed. Taken together, these data proved the safety and feasibility of iPC procedures in clinical translation.

## Discussion

Cardiovascular tissue engineering holds a great promise for heart regeneration and repair^33–36^. Among them, cardiac patch represents an excellent carrier to deliver stem cells and other therapeutic agents to the heart^10,27,37^. Yet, deployment of a cardiac patch normally requires open chest surgery. Minimally invasive delivery of cardiac patches has been reported before^6^. However, these procedures require special shape memory materials^38,39^. In addition, direct transplantation of cardiac patch to the epicardium disrupts the pericardium^6^, which is essential for cardiac homeostasis after MI^40^. In this study, we inject thermosensitive hydrogels (containing therapeutics) into the pericardial cavity. This is termed as intrapericardial or iPC injection. After injection, in situ gelation takes place and the hydrogel will form a cardiac patch-like structure in the pericardial cavity. This is very much like the “injection-molding” process used in the plastic industry. In this study we used iPS-CPCs and MSC-derived exosomes as two model therapeutics. The rationale behind that is we believe pluripotent stem cells and exosomes are the two most promising cell-based (derived) therapeutics for cardiac regeneration/repair, given most of the clinical trials on adult stem cells failed to produce functional benefits^41–43^.

iPS-CPCs are able to proliferate and differentiate into mature cardiomyocytes as well as the vascular lineages (endothelial cells and smooth muscle cells)^44–46^. In addition, paracrine activities of progenitor/stem cells are also major contributors to cardiac repair^47–49^. It has also been established that MSC-derived exosomes carrying proteins, nuclear acids, and other constituents are active players of paracrine activities^50^. Post-MI inflammation and cardiac remodeling can be modulated by exosomes treatment^48,51^. Exosomal transfer of miR-21, miR-125, miR-146, and other bioactive components improved cardiac repair by enhancing angiogenesis and cardiomyocyte survival^48,52^. Despite the encouraging results, poor survival caused by immune rejection and low retention rate have been the critical obstacles hindering clinical translation. Via iPC injection, we achieved higher cardiac retention of MSCs (Supplementary Fig. 13), MSC exosomes, and less immune response to iPS-CPCs.

Previous studies used non-hydrogel Gelfoam or saline to deliver adult stem cells, exosomes, or growth factors into the pericardial cavity to achieve cardiac repair^53–55^. Our studies used decellularized porcine heart ECM and MA–HA hydrogels as biomaterial carriers to deliver therapeutics to the pericardial cavity. Given the synergy between biomaterials and therapeutics in cardiac repair and the demonstrated feasibility of minimally invasive procedures to perform such interventions, our strategy represents an advancement to the field and offers knowledge regarding the safety and efficacy of iPC injection. In our study, decellularized porcine heart ECM and MA–HA hydrogels were used to deliver therapeutics via iPC injection, of which the ECM hydrogel is now used in clinical trials^56–58^. HA is the most abundant extracellular matrix component in the pericardium and was commonly used in biomedical studies by crosslinking with MA, an effective UV photoinitiator^59–61^. Therefore, the safety and translatability of these hydrogels can be foreseen.

Pericardial tamponade is a common medical emergency that is caused by the build up of fluid in pericardial cavity^62,63^. Under physiological conditions, balanced generation and drainage of pericardial fluid offer the heart with lubrication and protection. However, chest trauma, open chest surgery and other procedures break the balance and lead to tamponade^62^. In our study, we employed thermosensitive ECM hydrogel and pre-crosslinked HA hydrogel as the carriers of our therapeutics. In addition, minimally invasive iPC procedures preserve the intact pericardial structure. We did not record any tamponade events in any of our study rodents and pigs. It is noteworthy that iPC injection may not be the best choice for all injectable hydrogels. For example, a hydrogel with high swelling ability could potentially cause tamponade and pericardial effusion. Priority should be given to biocompatible, biodegradable, low swelling, conformable, and relatively soft hydrogels.

iPC injection of therapeutics can be performed in humans in a way similar to the standard LARIAT procedure^64^. During the LARIAT procedure, a local anesthetic is used to numb the procedure area. After the area is numbed, catheters are advanced into the pericardial space. Compared to the NOGA mapping guided trans-endomyocardial injection, which has been challenging, expensive, and needs special instruments, iPC injection can be performed under a fluoroscope which is universally available in most of the cardiovascular medicine units worldwide.

Overall, our studies demonstrated the safety, efficacy, and clinical feasibility of iPC injection of therapeutics for cardiac repair. iPC injection can be performed by an experienced cardiologist in a fairly short period of time and only conscious sedation is needed^65^. We confirmed that the technique is versatile as it can be used to deliver a variety of different therapeutics using various types of biomaterials. The delivery can achieve an ideal biodistribution in the myocardium while not causing safety concerns. Given clinical trials on cardiac regeneration are currently hindered by the lack of delivery efficiency, our results suggest iPC injection can be further developed as a new route for therapeutic administration (Supplementary Table 2). The goal of our study is to show iPC injection could be a method to effectively deliver multiple therapeutics to the heart. Future mechanistic and translational studies are needed to further develop those individual therapies.

## Methods

### Preparation of ECM hydrogel

ECM hydrogel was prepared accordingly^58^. Briefly, heart tissues were cut into pieces of 2 mm in thickness, followed by rinsing with deionized water (DI) water. Decellularization was performed by immersing tissues in 1% SDS in PBS for 4–5 days, until the tissue was white, then the tissues were placed in 1% Triton X-100 and stirred for 30 min for final cell removal. After that, the decellularized heart tissues were washed with DI water for more than 24 h to remove detergents. To produce the ECM hydrogel, the decellularized ECM was lyophilized and milled into a fine powder. After that, enzymatic digestion was performed using pepsin dissolved in 0.1 M HCl for at least 48 h (pepsin–matrix ratio at 1:10) with continuous stirring during the digestion. Finally, we adjusted the pH to 7.4 with NaOH on ice and diluted the ECM solution to 6 mg/mL. Gelling could occur at 37 °C water bath. Successful decellularization was confirmed with hematoxylin and eosin (H&E) staining after cryosectioning.

### iPS-CPC culture

iPS-CPCs were purchased from STEMCELL Technologies (iCell® Cardiac Progenitor Cells, 01279). To trace the iPS-CPCs in vivo, GFP transfection of iPS-CPC was performed using a transfection kit (Vigene Biosciences, CV10009).

### Preparation of MA–HA hydrogel

MA–HA hydrogel was prepared as previous described^38^. Briefly, 0.1 g HA was dissolved in 10 mL deionized water (DI) water, and stirred for 30 min. After that, 2 mL of 1 N NaOH as well as 0.5 mL MA was added into the solution and stirred for another 2 h. After that the mixture was placed at 4 °C for 24 h, followed by precipitation and purification with 95% ethanol. The lyophilized powder was then dissolved in pure water and dialyzed with a 12 kDa cellulose bag. Gelatin could occur with UV irradiation at a power of 4.5 mW/cm^2^ for 10 s.

### MSC culture and isolation of exosomes

Mesenchymal stem cells were purchased from the American Type Culture Collection (ATCC, VA, USA). After three passages, MSCs were cultured in serum-free Iscove’s modified Dulbecco’s medium (IMDM) for 48 h. The conditioned medium was collected and exosomes were isolated by the ultrafiltration method with a 0.22-μm filter. Transmission electron microscopy (TEM) was performed to confirm the morphology of exosomes. For TEM, exosomes were fixed with 4% paraformaldehyde (PFA) and 1% glutaraldehyde at room temperature.

### Rodent model of MI and iPC injection

Animal works were approved by Institute Animal Care and Use Committee (IACUC) of North Carolina State University (protocol#: 19-811-B), and all the procedures were complied with the ethical regulations. Mouse and rat are housed with 12 h light/12 h dark cycle at the temperature of 25 °C with 40–60% humidity. The rodent model of MI was induced as previously described^8^. Briefly, the animal was anesthetized through IP injection of ketamine-xylazine (KX) at a dose of 100 and 5 mg/kg, respectively, followed by ventilation, and thoracotomy. Then the left anterior descending coronary artery was ligated with a 6-0 suture while the pericardium was preserved. Infarction was confirmed by a pale color of the apex area. Immediately after MI, hydrogels with or without therapeutics were injected carefully into the pericardial cavity. The injection volumes were 100 µL (iPS-CPCs, rat) or 20 µL (Exosomes, mouse), respectively. As controls, we also performed IM injection in a single site located near the infarct zone. After injection, the chest was closed and the animal was allowed to recovery.

### Exosomes labelling and live imaging

To trace the in vivo biodistribution of exosomes, 10 μM DiD (Thermo Fisher Scientific, V22887) was used to label the exosomes. Exosomes in 20 μL PBS was injected as controls. The total exosome dose was 10 mg/mL in terms of the protein concentration.

### Cardiac function assessment

Cardiac function was measured at indicated time points. After anesthesia with inhalation of isoflurane, the animals were fixed to the operating plate with the body temperature maintained at 37 °C. Then the M-mode cardiac movement was collected and recorded with an echocardiography machine equipped with a 40 MHz transducer (Prospect T1, S-Sharp, Taiwan). Left ventricular dimensions at both diastole and systole were measured, and accordingly, the values of ejection fraction, fraction shortening, and left ventricular volume at end diastole and systole were calculated. Five continuous cardiac cycles were collected for each animal.

### Histological analysis

At indicated time points, animals were scarified with inhalation of CO_2_, followed by intraventricular perfusion of chilled saline and 4% PFA. With carefully dissection, the heart wrapped with pericardium was harvested and then immersed in 4% PFA overnight. After washing with PBS for twice, the heart was placed into 30% glucose, followed by embedding with OCT and cryosectioning. A series of sections in 5 µm thickness were collected and stored at −20 °C until use. H&E staining and Masson’s trichrome staining were performed following a standard protocol established in our lab.

### Immunocytochemistry

Cells were fixed with 4% PFA for 15 min at room temperature (RT), followed by PBS washing twice. Then the blocking serum was added and incubated at RT for 1 h to block the non-specific staining. After that the primary antibody was added and incubated overnight at 4 °C. After washing with PBS, the corresponding secondary antibody was incubated. DAPI was used to stain the nucleus. Terminal deoxynucleotidyl transferase dUTP nick end labeling (TUNEL) staining was introduced by using the commercial labelling kit (Promega, G3250), and after reaction, α-SA staining was performed.

### Intrapericardial injection in pigs

All animal procedures were approved by Institute Animal Care and Use Committee (IACUC) of North Carolina State University (protocol#: 20-137-B). All the procedures were complied with the ethical regulations. Male pigs (20–30 kg) were sedated with TKX cocktail (1 mL/13–30 kg IM). An ear venous catheter was placed once unconscious, and anesthesia was induced with isoflurane (up to 5% by mask). Then, the animal was intubated, and anesthesia was maintained with a mixture of isoflurane (2% in 100% oxygen). Sterile techniques were performed including sterile instruments, gloves, cap, and mask, sterile preparation of the skin and techniques to maintain sterility of the instruments during surgery. The pigs were placed in a supine position with a 30° inclination on the right side and left chest was used for port access. Local anesthesia was provided at the port sites by using an infusion of lidocaine or bupivacaine (1–2 mg/kg). Two 10 mm ports introduced with trocars were used for iPC injection needle and camera port. The camera port was placed in the third intercostal space at the level of scapula angle, while the injection port was placed in the seventh intercostal space at the posterior axillary line. After having access to the iPC cavity through the port, treatments were given through iPC injection by using a 15 cm introducer needle. The injection volume was 6 mL for each pig. After injection, the incision was closed.

### Blood test in pigs

Before and three days after injection, blood samples were drawn and blood test was performed by the Department of Clinical Pathology, College of Veterinary Medicine, NC State University.

### Pericardial fluid collection and inflammation assay

Pericardial fluid was collected before and 3 days after injection, and the levels of inflammatory cytokines in the pericardial fluid were measured by using the Porcine Cytokine Array (Raybiotech Inc, C1 Kit).

### Antibodies

Antibodies against Ki67 (ab16667, Abcam, 1:200), α-Sarcomeric Actinin (SA, ab9465, Abcam, 1:200), vWF (ab6994, Abcam, 1:100), CD31 (ab28364, Abcam, 1:100), Podoplanin (ab10288, Abcam, 1:500), Vimentin (ab92547, Abcam, 1:500), Sca-1 (ab109211, Abcam, 1:200), α-SMA (ab32575, Abcam, 1:500), MPO (PA5-16672, Thermo Fisher, 1:100), CD4 (ab237722, Abcam, 1:200), CD8 (ab33786, Abcam, 1:100), cTnT (MS-295P, Invitrogen, 1:100), Nkx2.5 (ab106923, Abcam, 1:100) as well as Alexa Fluo 594 or 488 conjugated Goat anti Rabbit or mouse secondary antibodies (1:500) were purchased from Abcam. TUNEL staining kit was purchased from Promega (G3250).

### Data acquisition and statistical analysis

Animals were randomized to treatment groups. Data acquisition and analysis were performed by investigators blind to the groups. Relevant datasets were acquired and proceeded with Excel 2016, Image J version 1.8.0, Living image 4.5 (Perkin Elmer), FV31S-SW viewer version 2.4 (Olympus), Echo Rebel version 5.3 (Echo), Prospect T-1 Version 3.132.2094 (S-Sharp). Statistical analysis was performed using GraphPad Prism 9 (Version 9.0.0.121) and data were expressed as mean ± SD. Comparisons between two groups were performed with unpaired, two-sided Student’s *t*-test, while for multiple group comparison, one-way ANOVA and two-way ANOVA was used with Bonferroni post correction. *p* < 0.05 was used as the criterion for significance.

### Reporting summary

Further information on research design is available in the Nature Research Reporting Summary linked to this article.

## Supplementary information

Supplementary Information

Description of Additional Supplementary Files

Supplementary Movie 1

Supplementary Movie 2

Supplementary Movie 3

Supplementary Movie 4

Supplementary Movie 5

Reporting Summary

## Data Availability

The data that support the findings of this study are available from the corresponding author upon reasonable request. Source data are provided with this paper.

## Source data

Source Data
