# Supplementary material for: Minimally invasive delivery of therapeutic agents by hydrogel injection into the pericardial cavity for cardiac repair
Source: Nat Commun. 2021 Mar 3;12:1412. doi: 10.1038/s41467-021-21682-7 (PMC7930285; doi:10.1038/s41467-021-21682-7)
Supplement: Supplementary file 1 — Supplementary Information [file 41467_2021_21682_MOESM1_ESM.pdf]

Supplementary information for

Minimally invasive delivery of therapeutic agents by hydrogel injection into the pericardial cavity for cardiac repair

Dashuai Zhu[1,2,5], Zhenhua Li[1,2,5], Ke Huang[1,2], Thomas Caranasos[3], Joseph Rossi[4], Ke Cheng[1,2,*]

Affiliations:

[1] Department of Molecular Biomedical Sciences and Comparative Medicine Institute, North Carolina State University, Raleigh, NC 27606, USA.

[2] Joint Department of Biomedical Engineering, University of North Carolina at Chapel Hill & North Carolina State University, Raleigh, NC 27606, USA.

[3] Division of Cardiothoracic Surgery, University of North Carolina at Chapel Hill, Chapel Hill, NC 27599, USA.

[4] Division of Cardiology, University of North Carolina at Chapel Hill, Chapel Hill, NC 27599, USA.

[5] These authors contributed equally to this work.

*Correspondence to: ke_cheng@ncsu.edu

Supplementary Figures

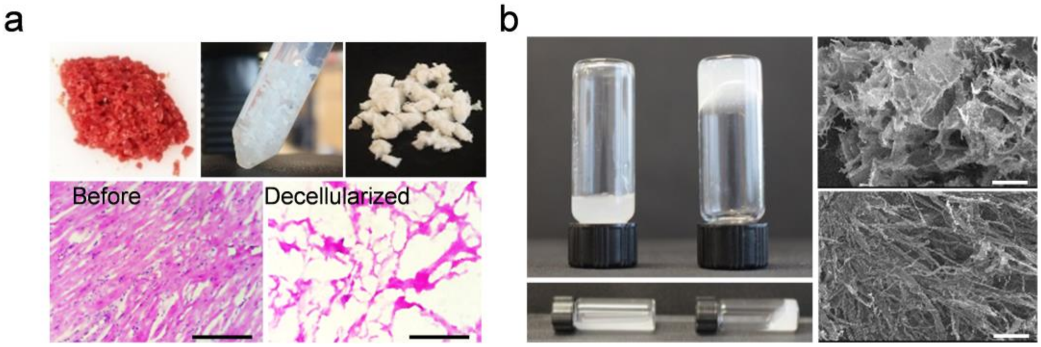

Supplementary Fig. 1. Preparation and characterization of ECM hydrogel. (a) Preparation of ECM hydrogel (up panel left to the right, fresh heart tissues, decellularized heart tissues and lyophilized decellularized heart tissues), and H-E staining for the confirmation of successful decellularization (bottom panel). Scale bar, 100μm. (b) In vitro gelation of ECM solution at 37℃ (left panel) and representative SEM images showing the distinct structure of ECM hydrogel (right panel, up: before gelation, bottom: after gelation). Scale bar, 100μm. This study was repeated three times independently with the similar results obtained.

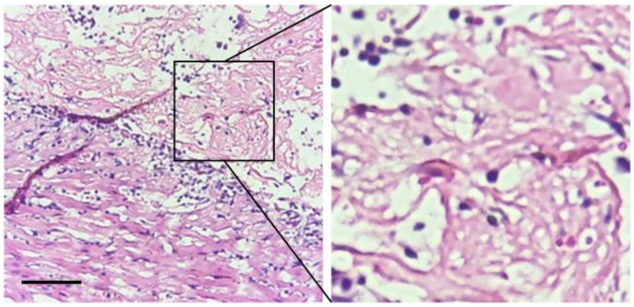

Supplementary Fig. 2. Biocompatibility of ECM hydrogel after injection into the pericardial cavity. ECM hydrogel was directly injected into pericardial cavity. 3 days later, H&E staining was performed for the evaluation of inflammatory infiltration. Scale bar, 100 μm. This study was repeated in three rats and similar results were obtained.

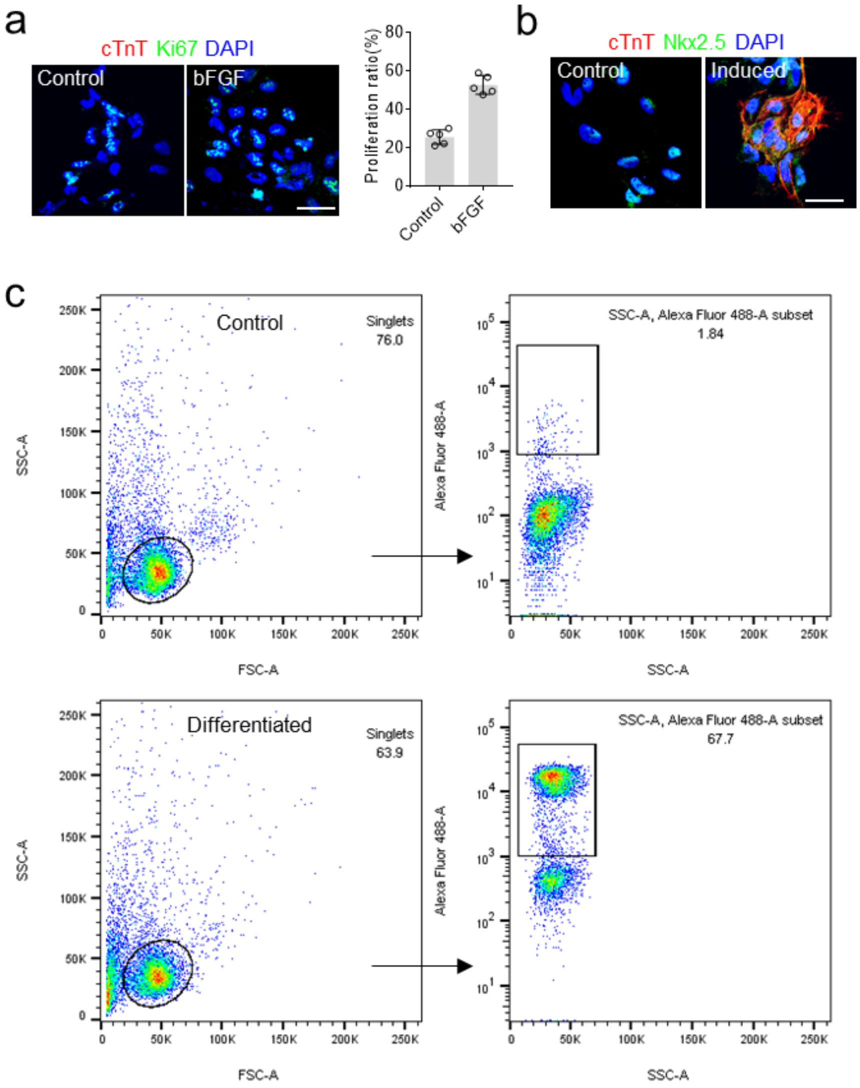

Supplementary Fig. 3. In vitro proliferation and differentiation of iPS-CPCs. (a) In vitro proliferation of iPS-CPCs when incubated with bFGF. Data were expressed as mean ± SD, n=5 repeats in each group. Scale bar, 40μm. (b and c) In vitro differentiation of iPS-CPCs into cardiomyocytes. b, immunofluorescence staining of induced cardiomyocyte differentiation from iPS-CPCs. Scale bar, 40μm. c, Flowcytometry confirming cardiomyocyte differentiation. cTnT was used as the marker of cardiomyocyte, and the corresponding secondary antibody was conjugated with Alexa Fluor 488. The ratio of Alexa Fluor 488 positive cells was determined. This study was repeated three times independently with similar results obtained. Source data are provided as a Source Data file.

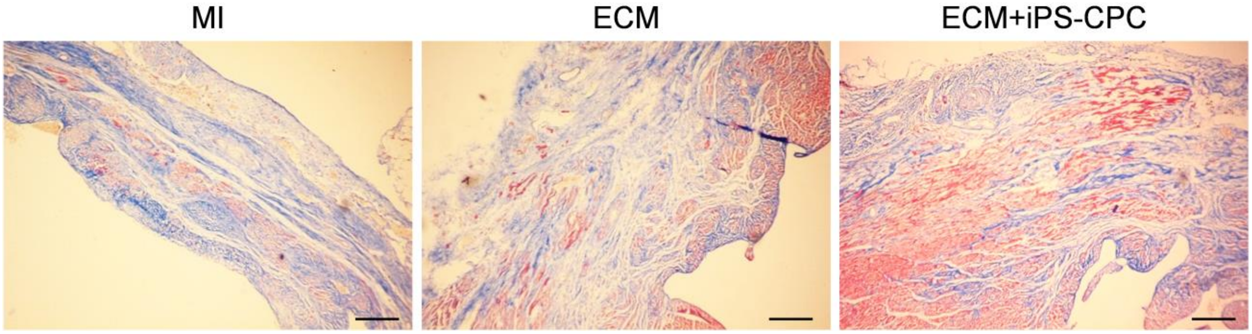

Supplementary Fig. 4. Representative Masson's trichrome-stained heart sections 4 weeks after treatment. 4 weeks after treatment, heart tissues were collected and stained with Masson's trichrome, the fibrotic scar was stained in blue, and viable myocardium was stained in red. After treatment, the cardiac morphology was improved. Scale bar, 100 μm. This study was repeated with four rats in each group and similar results were obtained.

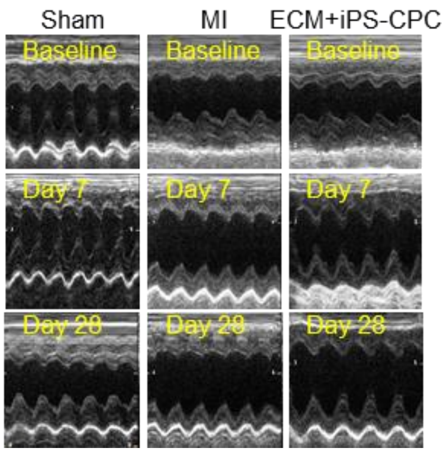

Supplementary Fig. 5. Echocardiography determination of cardiac functions. Representative M-mode images of left ventricle at indicated time points. Baseline cardiac functions were measured 2 hours after surgery.

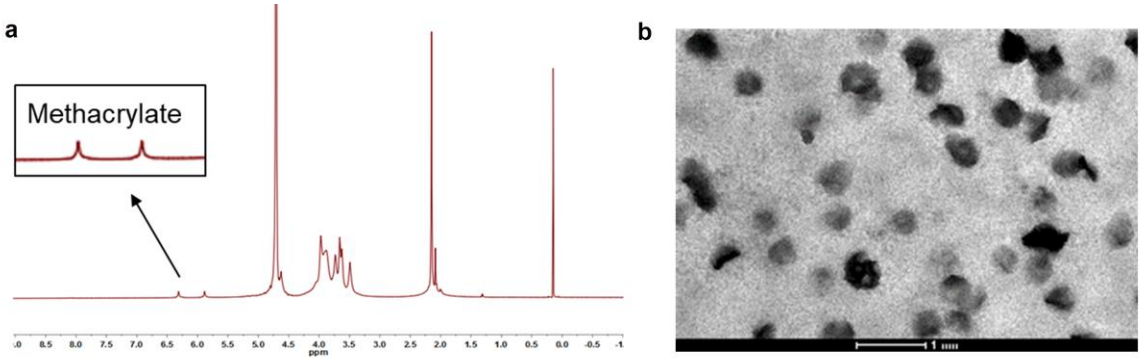

Supplementary Fig. 6. Characterization of MA-HA and exosomes. The MA modication of HA hydrogel was confirmed by mass spectrometry(a). Exosome morphology was confirmd with TEM (b). Scale bar, 1μm. TEM observation was repeated three times independently with similar results obtained.

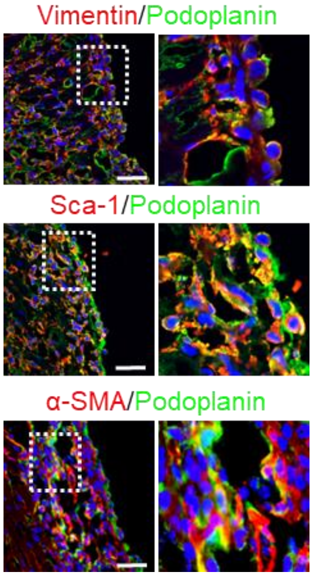

Supplementary Fig. 7. iPC injection of exosomes promotes EPDC differentiation. Expressions of stem/progenitor and stromal cell markers in epicardial cells after intrapericardial exosome injection. Scale bar, 60μm. This study was repeated with six mice with similar results obtained.

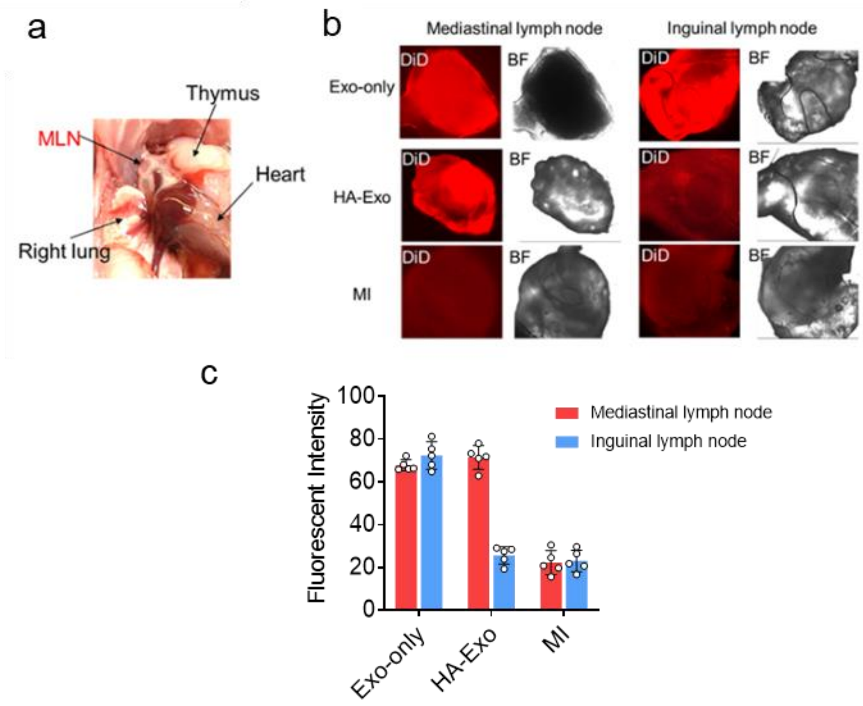

Supplementary Fig. 8. iPC-injected exosomes are observed in the lymph nodes. After iPC injection of DiD-labeled exosomes, an accumulation of exosomes was found in the cardiac-draining mediastinal lymph nodes (MLN) and inguinal lymph nodes (ILN). Delivery of exosome in HA hydrogel reduced exosome loss to the lymph nodes. Data are expressed as mean ± SD, n = 5 animals per group. Source data are provided in a Source Data file.

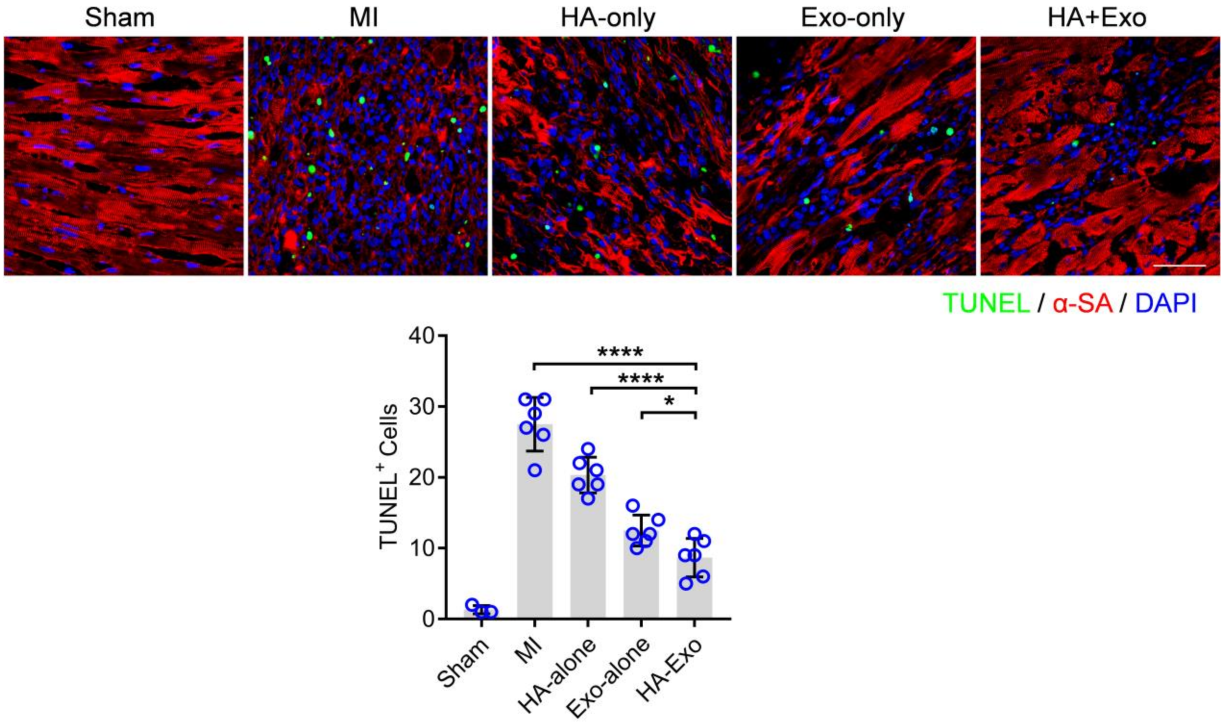

Supplementary Fig. 9. iPC injection of exosomes reduces apoptosis. Three days after therapy, TUNEL staining was performed to detect apoptotic cells and TUNEL positive cell numbers were counted. Scale bar, 100µm. Data are expressed as mean ± SD, n = 6 animals per group. Comparison between groups were performed with unpaired, two-sided student t-test. **p*<0.05, *****p*<0.0001. *p*=0.0226 for HA-Exo vs Exo-alone. Source data are provided in a Source Data file.

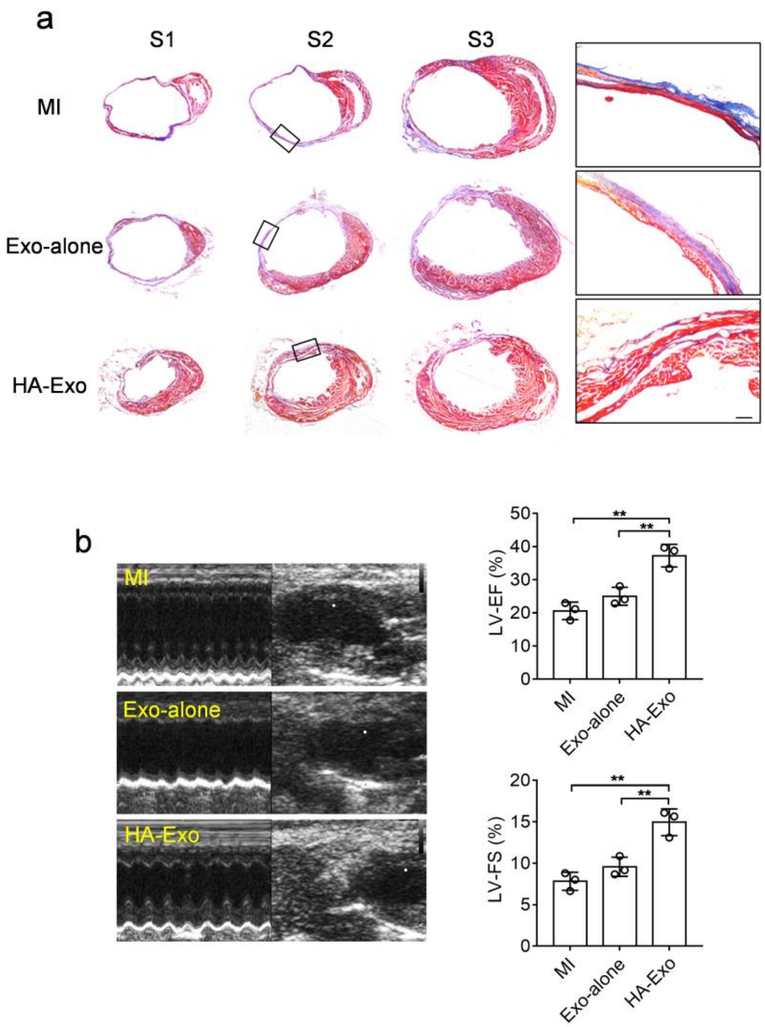

Supplementary Fig. 10. iPC injection of exosomes mitigates cardiac remodeling after MI. 3 months after iPC injection of exosomes in HA hydrogel, Masson's trichrome staining (a) revealed larger wall thickness and less scar in the treated animals. Scale bar, 100μm. (b) Echocardiography indicated augmented cardiac functions. All Data are expressed as mean ± SD, n = 3 animals per group. Comparison between groups were performed with unpaired, two-sided, one way ANOVA, ***p*<0.01. LV-EF: *p*=0.0011 for HA-Exo vs MI, *p*=0.0053 for HA-Exo vs Exo-alone. LV-FS: *p*=0.0013 for HA-Exo vs MI, *p*=0.0056 for HA-Exo vs Exo-alone. Source data are provided in a Source Data file.

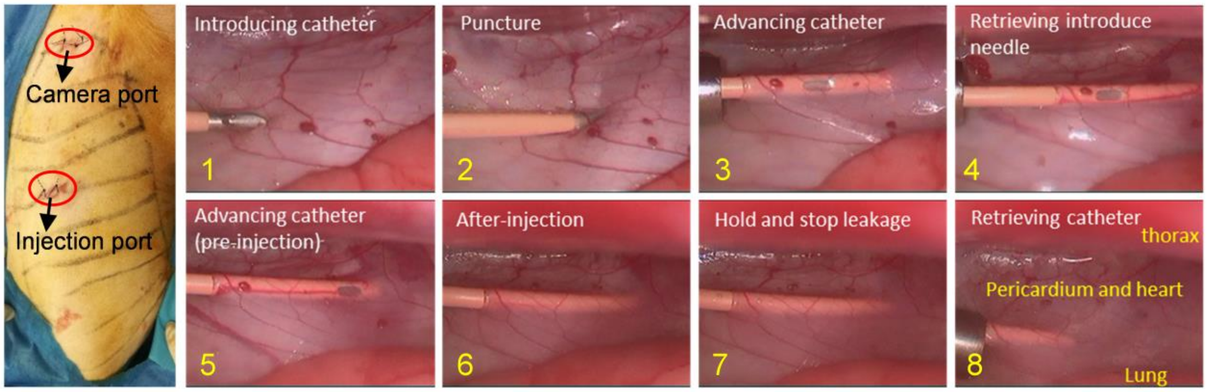

Supplementary Fig. 11. Minimally invasive iPC injection in pigs. Stilled images taken during the iPC injection procedures in pigs.

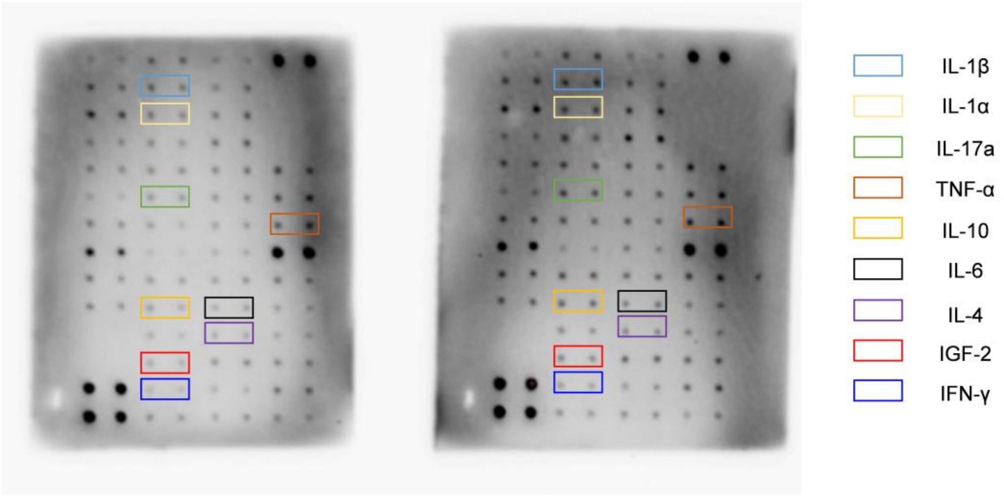

Supplementary Fig. 12. Cytokines array analysis of inflammation in pericardial fluid after iPC injection in pigs. Pericardial fluid was harvested before and after iPC injection for inflammatory cytokine analysis. This study was performed with three pigs and similar results were obtained.

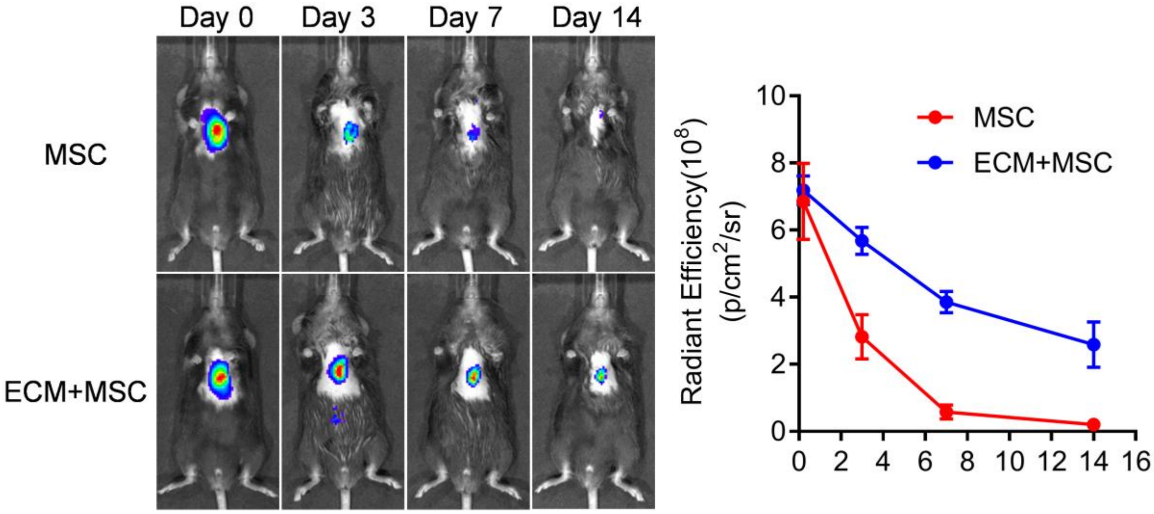

Supplementary Fig. 13. iPC injection in ECM hydrogel enhances the cardiac retention of mesenchymal stem cells (MSCs). Live image was performed to detect the retention of injected cells at days 0, 3, 7 and 14 after injection(left), and accordingly, the fluorescence signal intensity was measured(right). Data were expressed as mean ± SD, n=3 animals in each group. Source data are provided in a Source Data file.

Supplementary Table 1. Diameters of left ventricular at both end-diastole (LVIDd) and end-systole(LVIDs) in rats

	Baseline		Day 7		Day 28	
	LVIDd(cm)	LVIDs(cm)	LVIDd(cm)	LVIDs(cm)	LVIDd(cm)	LVIDs(cm)
Sham	0.654±0.017	0.343±0.026	0.679±0.016	0.366±0.008	0.707±0.007	0.373±0.009
MI	0.648±0.026	0.504±0.006	0.724±0.005	0.608±0.011	0.845±0.009	0.744±0.007
ECM	0.658±0.013	0.516±0.017	0.704±0.023	0.575±0.016	0.775±0.012	0.654±0.012
ECM+iPS-CPC	0.659±0.027	0.517±0.032	0.696±0.027	$0.554\pm0.028^{*}$	$0.729\pm0.023^{**}$	$0.586\pm0.006^{**}$

Data were expressed as mean ± SD, n=4 per group, *p<0.05 vs MI, **p<0.01 vs MI or ECM

Supplementary Table 2. Comparison of various administration routes to the heart.

	Intravenous (IV)	Intracoronary (IC)	Intramyocardial (IM)	Cardiac patch	Intrapericardial (iPC)
Easy to perform	Yes	Yes, needs cath lab and interventional cardiologist	No, requires open-chest Suegery, unless use NOGA-endomyocardial injection	No, requires open-chest surgery	Yes
Invasiveness	Low	Low	High, unless use NOGA-endomyocardial injection	High	Low
Retention in the heart	None to low	Low	Medium	High	High
Cost	Low	Medium	High	High	Low
