# Supplementary material for: Minimally invasive delivery of therapeutic agents by hydrogel injection into the pericardial cavity for cardiac repair
Source: Nat Commun. 2021 Mar 3;12:1412. doi: 10.1038/s41467-021-21682-7 (PMC7930285; doi:10.1038/s41467-021-21682-7)
Supplement: Supplementary file 2 — Description of Additional Supplementary Files [file 41467_2021_21682_MOESM2_ESM.pdf]

Description of Additional Supplementary Files

File Name: Supplementary Movie 1.
Description: iPC injection in a mouse model of myocardial infarction (MI). After induction of MI model by LAD ligation, iPC injection was performed with a 0.3 mL syringe with a low angle puncture into the pericardium. The injection volume is 20 μL.

File Name: Supplementary Movie 2.
Description: iPC injection in a rat model of myocardial infarction (MI). After induction of MI model by LAD ligation, iPC injection was performed with a 0.3 mL syringe with a low angle puncture into the pericardium. A blue dye was employed to confirm the contents were injected into the pericardial cavity but not into the myocardium. The injection volume was 50 μL.

File Name: Supplementary Movie 3.
Description: Minimally invasive iPC injection in porcine with 2 incisions. To access to the pericardial cavity of pigs, two trocars were placed at the 3rd and the 7th intercostal for camera and injection catheter entry respectively. iPC injection was performed with a puncture into the pericardial cavity with a 16G catheter. The injection volume is 5 mL in pigs.

File Name: Supplementary Movie 4.
Description: Representative confocal Z-stack showing in vivo differentiation of iPS-CPCs into cardiomyocytes 4 weeks after iPC injection. iPS-CPCs were tagged with GFP, and cardiomyocytes were marked with α-sarcomeric actinin (α-SA) in red.

File Name: Supplementary Movie 5.
Description: Representative confocal Z-stack confirming epicardial uptake of exosomes after iPC injection. Exosomes were pre-labeled with DiD (red), and epicardium was labeled with podoplanin (green).
