# Supplementary material for: Minimally invasive delivery of therapeutic agents by hydrogel injection into the pericardial cavity for cardiac repair
Source: Nat Commun. 2021 Mar 3;12:1412. doi: 10.1038/s41467-021-21682-7 (PMC7930285; doi:10.1038/s41467-021-21682-7)
Supplement: Supplementary file 8 — Reporting Summary [file 41467_2021_21682_MOESM8_ESM.pdf]

nature research

Corresponding author(s): Ke Cheng

Last updated by author(s): Jan 18, 2021

# Reporting Summary

Nature Research wishes to improve the reproducibility of the work that we publish. This form provides structure for consistency and transparency in reporting. For further information on Nature Research policies, see our Editorial Policies and the Editorial Policy Checklist.

## Statistics

For all statistical analyses, confirm that the following items are present in the figure legend, table legend, main text, or Methods section.

| n/a | Confirmed | |
|---|---|---|
| ☐ | ☒ | The exact sample size (*n*) for each experimental group/condition, given as a discrete number and unit of measurement |
| ☐ | ☒ | A statement on whether measurements were taken from distinct samples or whether the same sample was measured repeatedly |
| ☐ | ☒ | The statistical test(s) used AND whether they are one- or two-sided<br>*Only common tests should be described solely by name; describe more complex techniques in the Methods section.* |
| ☒ | ☐ | A description of all covariates tested |
| ☐ | ☒ | A description of any assumptions or corrections, such as tests of normality and adjustment for multiple comparisons |
| ☐ | ☒ | A full description of the statistical parameters including central tendency (e.g. means) or other basic estimates (e.g. regression coefficient) AND variation (e.g. standard deviation) or associated estimates of uncertainty (e.g. confidence intervals) |
| ☐ | ☒ | For null hypothesis testing, the test statistic (e.g. *F*, *t*, *r*) with confidence intervals, effect sizes, degrees of freedom and *P* value noted<br>*Give P values as exact values whenever suitable.* |
| ☒ | ☐ | For Bayesian analysis, information on the choice of priors and Markov chain Monte Carlo settings |
| ☒ | ☐ | For hierarchical and complex designs, identification of the appropriate level for tests and full reporting of outcomes |
| ☒ | ☐ | Estimates of effect sizes (e.g. Cohen's *d*, Pearson's *r*), indicating how they were calculated |

*Our web collection on statistics for biologists contains articles on many of the points above.*

## Software and code

Policy information about availability of computer code

| | |
|---|---|
| Data collection | Excel 2016(Microsoft), Image J version 1.8.0, Living Image 4.5 (Perkin Elmer), FV31S-SW viewer version 2.4 (Olympus), Echo Rebel version 5.3 (ECHO), Prospect T-1 Version 3.132.2094(S-Sharp) |
| Data analysis | Live imaging data can be acquired directly with the software; Cardiac function measurements were acquired with Prospect T-1 to generate statistical data; Histological staining images were acquired with FV-31S-SW software and ECHO software and analyzed by Image J to generate statistical data. All the statistical datasets were analyzed using GraphPad Prism 9(Version 9.0.0.121). |

For manuscripts utilizing custom algorithms or software that are central to the research but not yet described in published literature, software must be made available to editors and reviewers. We strongly encourage code deposition in a community repository (e.g. GitHub). See the Nature Research guidelines for submitting code & software for further information.

## Data

Policy information about availability of data

All manuscripts must include a data availability statement. This statement should provide the following information, where applicable:

- Accession codes, unique identifiers, or web links for publicly available datasets
- A list of figures that have associated raw data
- A description of any restrictions on data availability

All data from this study is available from the corresponding author upon reasonable request.



April 2020

# Field-specific reporting

Please select the one below that is the best fit for your research. If you are not sure, read the appropriate sections before making your selection.

- [x] Life sciences
- [ ] Behavioural & social sciences
- [ ] Ecological, evolutionary & environmental sciences

For a reference copy of the document with all sections, see nature.com/documents/nr-reporting-summary-flat.pdf

# Life sciences study design

All studies must disclose on these points even when the disclosure is negative.

| | |
|---|---|
| Sample size | No statistical methods were used to predetermine the sample size. For all these experiments, at least 3 independent repeats/animals were deployed in each group, which allowed sufficient statistics to perform unpaired student t-test or ANOVA analysis, and gave p values to indicate the significance. To proceed with statistical analysis, more than three animals or repeats were performed. |
| Data exclusions | No data was excluded. |
| Replication | All experiments were repeated with at least three independent tests, and all attempts at replication were successful. |
| Randomization | All samples was randomly assigned, and analyzed together in each experiment. |
| Blinding | Data acquisition and analysis were performed by investigators who are blinded to the groups. |

# Reporting for specific materials, systems and methods

We require information from authors about some types of materials, experimental systems and methods used in many studies. Here, indicate whether each material, system or method listed is relevant to your study. If you are not sure if a list item applies to your research, read the appropriate section before selecting a response.

## Materials & experimental systems

| n/a | Involved in the study | |
|---|---|---|
| [ ] | [x] | Antibodies |
| [ ] | [x] | Eukaryotic cell lines |
| [x] | [ ] | Palaeontology and archaeology |
| [ ] | [x] | Animals and other organisms |
| [x] | [ ] | Human research participants |
| [x] | [ ] | Clinical data |
| [x] | [ ] | Dual use research of concern |

## Methods

| n/a | Involved in the study | |
|---|---|---|
| [x] | [ ] | ChIP-seq |
| [ ] | [x] | Flow cytometry |
| [x] | [ ] | MRI-based neuroimaging |

## Antibodies

Antibodies used

Ki67 (rabbit, ab16667, Abcam, 1:200, https://www.abcam.com/ki67-antibody-sp6-ab16667.html),
α- Sarcomeric Actinin (Mouse, SA, ab9465, Abcam, 1:200, https://www.abcam.com/sarcomeric-alpha-actinin-antibody-ea-53-ab9465.html),
vWF (Rabbit, ab6994, Abcam, 1:100, https://www.abcam.com/von-willebrand-factor-antibody-ab6994.html),
CD31 (Rabbit, ab28364, Abcam, 1:100, https://www.abcam.com/cd31-antibody-ab28364.html),
Podoplanin (Mouse, ab10288, Abcam, 1:500, https://www.abcam.com/podoplanin--gp36-antibody-18h5-ab10288.html),
Vimentin (Rabbit, ab92547, Abcam, 1:500, https://www.abcam.com/vimentin-antibody-epr3776-cytoskeleton-marker-ab92547.html),
Sca-1 (Rabbit, ab109211, Abcam, 1:200, https://www.abcam.com/sca1--ly6ae-antibody-epr3355-ab109211.html),
α-SMA (Rabbit, ab32575, Abcam, 1:500, https://www.abcam.com/alpha-smooth-muscle-actin-antibody-e184-ab32575.html),
MPO (Rabbit, PA5-16672, Thermo Fisher, 1:100, https://www.thermofisher.com/antibody/product/PA5-16672.html),
CD4 (Rabbit, ab237722, Abcam, 1:200, https://www.abcam.com/cd4-antibody-cal4-ab237722.html),
CD8 (Mouse, ab33786, Abcam, 1:100, https://www.abcam.com/cd8-alpha-antibody-ox-8-ab33786.html),
cTnT (Mouse, MS-295P, Invitrogen, 1:100, http://tools.thermofisher.com/content/sfs/brochures/D11736~.pdf),
Nkx2.5 (Goat, ab106923, Abcam, 1:100, https://www.abcam.com/nkx25-antibody-ab106923.html)
Alexa Fluo 594 or 488 conjugated Goat anti Rabbit or mouse secondary antibodies(ab150077, ab150080, ab150113, ab150116, 1:500) and Alexa Fluor-488 conjugated Donkey anti-Goat(ab150129), Alexa Fluor-594 conjugated Donkey anti-mouse(ab150108) (1:500) were purchased from Abcam.
TUNEL staining kit was purchased from Promega (G3250).

Validation

All antibodies were purchased after referring to the manufacturer's website.
Ki67 (rabbit, ab16667, Abcam, 1:200, https://www.abcam.com/ki67-antibody-sp6-ab16667.html),

α-Sarcomeric Actinin (Mouse, SA, ab9465, Abcam, 1:200, https://www.abcam.com/sarcomeric-alpha-actinin-antibody-ea-53-ab9465.html),
vWF (Rabbit, ab6994, Abcam, 1:100, https://www.abcam.com/von-willebrand-factor-antibody-ab6994.html),
CD31 (Rabbit, ab28364, Abcam, 1:100, https://www.abcam.com/cd31-antibody-ab28364.html),
Podoplanin (Mouse, ab10288, Abcam, 1:500, https://www.abcam.com/podoplanin--gp36-antibody-18h5-ab10288.html),
Vimentin (Rabbit, ab92547, Abcam, 1:500, https://www.abcam.com/vimentin-antibody-epr3776-cytoskeleton-marker-ab92547.html),
Sca-1 (Rabbit, ab109211, Abcam, 1:200, https://www.abcam.com/sca1--ly6ae-antibody-epr3355-ab109211.html),
α-SMA (Rabbit, ab32575, Abcam, 1:500, https://www.abcam.com/alpha-smooth-muscle-actin-antibody-e184-ab32575.html),
MPO (Rabbit, PA5-16672, Thermo Fisher, 1:100, https://www.thermofisher.com/antibody/product/PA5-16672.html),
CD4 (Rabbit, ab237722, Abcam, 1:200, https://www.abcam.com/cd4-antibody-cal4-ab237722.html),
CD8 (Mouse, ab33786, Abcam, 1:100, https://www.abcam.com/cd8-alpha-antibody-ox-8-ab33786.html),
cTnT (Mouse, MS-295P, Invitrogen, 1:100, http://tools.thermofisher.com/content/sfs/brochures/D11736~.pdf),
Nkx2.5 (Goat, ab106923, Abcam, 1:100, https://www.abcam.com/nkx25-antibody-ab106923.html)
Alexa Fluo 594 or 488 conjugated Goat anti Rabbit or mouse secondary antibodies(ab150077, ab150080, ab150113, ab150116, 1:500) and Alexa Fluor-488 conjugated Donkey anti-Goat(ab150129), Alexa Fluor-594 conjugated Donkey anti-mouse(ab150108) (1:500) were purchased from Abcam.
TUNEL staining kit was purchased from Promega (G3250).
By referring to their reactivity to mouse, rat, as well as application in immuno-fluoresence staining, flow-cytometry, these antibodies were ordered. All the antibodies used in this study work excellently.

# Eukaryotic cell lines

Policy information about cell lines

| | |
|---|---|
| Cell line source(s) | Mesenchymal stem cells purchased from the American Type Culture Collection (ATCC, VA, USA); Neonatal rat cardiomyocytes (NRCMs) were isolated in our lab according to Qiao, L., et al. J Clin Invest 129, 2237-2250 (2019); iPSC-CPC cells were purchased from STEMCELL Technologies. |
| Authentication | MSCs were tested for its specific marker (CD90+, CD105+ and CD3a-, CD34-, CD117-) by flowcytometry; NRCMs were positive for cardiomyocyte specific marker alpha-SA; iPSC-CPCs were positive for cardiac transcript factor Nkx2.5. Authentication of NRCMs and iPSC-CPCs were performed by immunofluorescence staining. |
| Mycoplasma contamination | Mycoplasma testing (PCR method) is routinely performed in the lab, and all cell lines tested negative for mycoplasma contamination. |
| Commonly misidentified lines (See ICLAC register) | No misidentified cell lines were used in the study. |

# Animals and other organisms

Policy information about studies involving animals; ARRIVE guidelines recommended for reporting animal research

| | |
|---|---|
| Laboratory animals | Male C57BL/6 mice and SD rats, at age of 8 weeks were purchased from Charles River Laboratory. Mouse and rat are housed with 12h light/12h dark cycle at the temperature of 25°C with 40-60% humidity. Male Yorkshire pigs, 8 weeks, were ordered from Unit II Palmetto. |
| Wild animals | No wild animals involved in this study. |
| Field-collected samples | This study did not involve field-collected samples. |
| Ethics oversight | All animal study was performed under guidelines of the North Carolina State University, Institutional Animal Care and Use Committee (IACUC), under approved IACUC # 19-811-B and 20-137-B. |

Note that full information on the approval of the study protocol must also be provided in the manuscript.

# Flow Cytometry

## Plots

Confirm that:

- [x] The axis labels state the marker and fluorochrome used (e.g. CD4-FITC).
- [x] The axis scales are clearly visible. Include numbers along axes only for bottom left plot of group (a 'group' is an analysis of identical markers).
- [x] All plots are contour plots with outliers or pseudocolor plots.
- [x] A numerical value for number of cells or percentage (with statistics) is provided.

## Methodology

| | |
|---|---|
| Sample preparation | iPSC-CPC cells were purchased from STEMCELL Technologies. Cells were acquired after digestion from culture, followed by |

| | |
|---|---|
| | two wash with PBS, primary antibody of cTnT were added and incubated overnight. After two wash with PBS, Alexa Fluor 488 labeled secondary antibody was added and incubated for an hour. Then the cells were fixed with 0.5% PFA for 15min at room temperature. |
| Instrument | BD LSR-II Flow Cytometer |
| Software | BD FACSDiva Software |
| Cell population abundance | The abundance of cTnT+ cells was about 67%. |
| Gating strategy | The detailed strategy was shown in supplementary information. FSC-A/SSC-A was used to gate singlets, followed by Alexa Fluor 488-A/SSC-A to gate cTnT+ cell populations. |

☒ Tick this box to confirm that a figure exemplifying the gating strategy is provided in the Supplementary Information.
